# Organizational Patient Safety Culture, Psychological Resilience, and Job Satisfaction Among Nurses in ISO-Certified and Non-ISO-Certified Departments: An Exploratory Cross-Sectional Comparison

**DOI:** 10.3390/healthcare14172789

**Published:** 2026-09-01

**Authors:** Georgios Manomenidis, Konstantinos Gkotsidis, Savvato Karavasileiadou, Vasiliki Georgousopoulou

**Affiliations:** 1 Department of Nursing, Democritus University of Thrace, 68100 Alexandroupoli, Greece; vageorgo@nurs.duth.gr; 2 Office of the Deputy Administrator, University Hospital of Alexandroupolis, 68100 Alexandroupolis, Greece; konstantinosgkotsidis@gmail.com; 3 Department of Community and Psychiatric Mental Health Nursing, College of Nursing, Princess Nourah bint Abdulrahman University, Riyadh 11671, Saudi Arabia

**Keywords:** patient safety culture, ISO certification, nurses, psychological resilience, job satisfaction, healthcare quality, quality management systems, hospital departments

## Abstract

**Highlights:**

**What are the main findings?**

- Nurses working in ISO-certified departments reported higher Staffing and Work Pace and Reporting Patient Safety Events scores, but lower Handoffs and Information Exchange scores, than nurses working in non-ISO-certified departments.
- Psychological resilience and job satisfaction did not differ in the unadjusted comparisons; however, after adjustment for demographic and professional characteristics, ISO certification was positively associated with job satisfaction but not with resilience.

**What are the implications of the main findings?**

- Working in ISO-certified departments showed selective and mixed associations with patient safety culture rather than a uniformly more favorable profile.
- The absence of consistent associations across all outcomes indicates that certification status alone does not account for nurses’ resilience, job satisfaction, or all dimensions of patient safety culture.

**Abstract:**

Background/Objectives: ISO-based quality-management systems may be associated with organizational processes relevant to patient safety, but evidence regarding nurses’ safety-culture perceptions, resilience, and job satisfaction remains limited. This study compared nurses working in ISO-certified and non-ISO-certified hospital departments. Methods: This cross-sectional study included 263 nurses from 29 departments (11 ISO-certified and 18 non-ISO-certified) in one tertiary hospital in Northern Greece. Patient safety culture was assessed using the 10 dimensions of the Hospital Survey on Patient Safety Culture 2.0, together with the Brief Resilience Scale and Job Satisfaction Index. Unadjusted comparisons and covariate-adjusted linear regression models with heteroscedasticity-robust standard errors were performed, with Benjamini–Hochberg false-discovery-rate correction. Results: In unadjusted analyses, nurses working in ISO-certified departments reported higher Staffing and Work Pace (MD = 0.222, 95% CI [0.094, 0.351], *g* = 0.40) and Reporting Patient Safety Events (MD = 0.427, 95% CI [0.134, 0.719], *g* = 0.37), but lower Handoffs and Information Exchange (MD = −0.295, 95% CI [−0.458, −0.131], *g* = −0.45); all three differences remained significant after false-discovery-rate correction. Resilience and job satisfaction did not differ in unadjusted analyses. After adjustment and correction, working in an ISO-certified department was associated with higher Staffing and Work Pace, Supervisor/Manager/Clinical Leader Support, and job satisfaction, and with lower Hospital Management Support and Handoffs and Information Exchange; no association with resilience was observed. Conclusions: Working in ISO-certified departments showed selective, modest, and mixed nurse-reported associations. Because certification was a department-level characteristic and within-department clustering could not be modeled, the findings should be interpreted as exploratory rather than as estimates of a department-level certification effect.

## 1. Introduction

Improving healthcare quality and ensuring patient safety have become essential priorities for healthcare systems worldwide. Despite significant advances in medical science and healthcare technology, preventable adverse events still present a major challenge, affecting millions of patients each year and resulting in unnecessary illness, death, and increased healthcare costs [1]. Modern patient safety approaches emphasize that safe care not only depends on healthcare providers’ competence, but also on organizational structures, leadership, communication, staffing, teamwork, and organizational culture [2,3,4]. Donabedian’s Structure Process Outcome model provides an overarching framework for understanding how organizational arrangements may shape care processes and subsequent outcomes [5]. In the adapted framework used in the present study, ISO certification was considered a department-level structural characteristic, patient safety culture a process-related organizational context, and psychological resilience and job satisfaction nurse-related outcomes. Among the most widely used structural quality-management frameworks are ISO 9001:2015 [6] and the healthcare-specific European standard EN 15224:2016 [7], which adapts the requirements of EN ISO 9001:2015 to healthcare organizations. These standards provide structured approaches to procedures, documentation, performance measurement, risk assessment, corrective action, and continuous improvement.

The adoption of ISO-based quality management systems has increased significantly in healthcare organizations in recent years. Evidence from management-system certification, hospital accreditation, and ISO 9001 implementation suggests associations with improved process standardization, documentation, risk-management practices, organizational efficiency, and selected quality outcomes; however, these effects vary across settings and depend on implementation quality [8,9,10]. Certification does not itself ensure favorable organizational outcomes, because its effects depend on implementation quality, leadership commitment, staff engagement, organizational readiness, and integration into daily clinical practice [10,11]. Within the adapted framework, standardized procedures, documented responsibilities, internal audits, corrective-action systems, formal incident-reporting processes, and systematic monitoring represent structural mechanisms that may shape patient safety culture through accountability, communication, staffing processes, organizational learning, and continuity of information [8,9,10,11,12]. Their relationships with resilience and job satisfaction are expected to be more indirect and may be offset by documentation burden, procedural rigidity, or compliance-related workload. Uniformly favorable associations across organizational processes and nurse-related outcomes were therefore not expected.

One crucial organizational factor linked to healthcare quality is the culture of patient safety. Patient safety culture reflects the shared values, beliefs, attitudes, perceptions, and behavioral norms that demonstrate an organization’s commitment to patient safety [13]. A positive safety culture is characterized by open communication, teamwork, mutual trust, strong leadership, organizational learning, adequate staffing, and non-punitive responses to errors [14].

Within the process-related component of the adapted framework, these dimensions indicate how formal organizational and quality-management arrangements are enacted and experienced in routine clinical practice. More favorable patient-safety-culture perceptions have been associated with better professional quality of life, higher job satisfaction, lower turnover intention, and selected safety-related outcomes, whereas job stress and unfavorable working conditions have been associated with less favorable perceptions [15,16,17,18,19]. Nurses are particularly relevant informants because they directly experience staffing arrangements, communication, handoffs, incident reporting, leadership practices, and the implementation of safety protocols in everyday care [2,14].

At the outcome level of the adapted framework, psychological resilience and job satisfaction represent distinct but related nurse outcomes. Resilience concerns individuals’ capacity to adapt and recover when exposed to adversity and has been associated among nurses with lower burnout, better mental health, stronger work engagement, and more favorable professional outcomes [20,21,22,23,24,25]. Job satisfaction reflects nurses’ overall evaluation of their work and is associated with leadership support, professional autonomy, staffing, recognition, workplace relationships, burnout, and turnover-related outcomes [19,26,27,28,29,30]. Both outcomes may be related to patient safety culture through supportive leadership, teamwork, communication openness, organizational learning, workload organization, psychological safety, and empowerment [28,31,32,33,34]. Direct associations between certification status and these nurse-related outcomes are also possible but may be weaker and dependent on implementation quality and maturity.

Against this Structure–Process–Outcome background, evidence remains limited regarding whether nurses working in ISO-certified departments report differences primarily in patient-safety-culture processes or whether such differences extend to nurse-related outcomes such as psychological resilience and job satisfaction. Previous research has largely evaluated accreditation or certification through organizational performance indicators, compliance measures, and quality-improvement processes [9,10]. In Greece, available research has examined ISO certification in relation to patient safety culture but has not simultaneously assessed safety-culture dimensions, psychological resilience, and job satisfaction [11]. Consequently, it remains unclear whether working in ISO-certified departments is associated primarily with selected safety-culture processes or whether such associations extend to broader psychosocial outcomes. Therefore, the present study aimed to com-pare individual nurses’ reported patient safety culture, psychological resilience, and job satisfaction according to the ISO-certification status of the departments in which they worked; to examine whether associations were more consistent for safety-culture dimensions than for psychosocial outcomes; and to assess the relationships of patient safety culture with resilience and job satisfaction. The estimand of interest was the nurse-level difference associated with working in an ISO-certified rather than a non-ISO-certified department, not an independent department-level causal effect of certification. Given the limited and mixed previous evidence, no formal directional hypotheses were prespecified, and the analyses were exploratory.

## 2. Materials and Methods

### 2.1. Study Design and Setting

An observational, comparative cross-sectional study was conducted in 29 departments of one tertiary hospital in Northern Greece between July and September 2025, comprising 11 ISO-certified and 18 non-ISO-certified departments. ISO-certification status was a shared department-level exposure, whereas the individual nurse was the unit of observation and analysis. Because the study was not designed as a multilevel department-based investigation, possible within-department dependence could not be incorporated into the analyses. This manuscript was reported in accordance with the Strengthening the Reporting of Observational Studies in Epidemiology (STROBE) guidelines for cross-sectional studies [35]. ISO certification was operationalized as a binary department-level variable based on departmental information. Detailed information on the specific ISO standard and version, certification scope, initial certification date, recertification history, audit findings, and implementation maturity was unavailable in the analytic dataset. Consequently, the exposure represented documented certification status rather than the degree or quality of quality-management-system implementation.

### 2.2. Sample and Data Collection

A total of 263 nurses participated: 165 nurses from 11 ISO-certified departments and 98 nurses from 18 non-ISO-certified departments. The departments were purposively selected to include both certification groups. Within these departments, eligible nurses who were accessible during the study period were recruited through convenience sampling because a complete sampling frame was unavailable to the research team. Nurses were eligible if they had been continuously employed in the institution for at least one year and were willing to participate voluntarily. Data was collected using paper-based, self-administered questionnaires during a predefined data-collection period. Before completing the questionnaire, participants received written information about the study purpose, voluntary participation, anonymity, confidentiality, and their right to withdraw at any time without consequences. Written informed consent was obtained from all participants. Clear instructions for questionnaire completion were provided, and completed questionnaires were returned in sealed envelopes or deposited in a sealed collection box to preserve anonymity. The total number of eligible nurses employed in the participating departments, as well as the numbers who received or declined the invitation, were not recorded. Consequently, a formal response rate could not be calculated. No formal a priori sample-size calculation was conducted; instead, the study sought to recruit the largest feasible sample of eligible nurses from the selected ISO-certified and non-ISO-certified departments during the predefined data-collection period.

### 2.3. Measurements

The survey tool included four sections: (a) sociodemographic characteristics, (b) the Hospital Survey on Patient Safety Culture (HSOPSC), (c) the Brief Resilience Scale (BRS), and (d) the Job Satisfaction Index (JSI).

#### 2.3.1. Socio-Demographic Characteristics

The demographic characteristics questionnaire was constructed for the purpose of the study and included questions about gender, age, level of education, marital status, years of experience, ISO certification, and type of department.

#### 2.3.2. Hospital Survey on Patient Safety Culture (HSOPSC)

Patient safety culture was assessed using the Agency for Healthcare Research and Quality Hospital Survey on Patient Safety Culture Version 2.0 (HSOPSC 2.0) [13]. A Greek-language version previously used in Greek hospital departments was administered [11]. The instrument comprises 32 items, allocated in 10 dimensions: Teamwork, Staffing and Work Pace, Organizational Learning–Continuous Improvement, Response to Error, Supervisor/Manager/Clinical Leader Support for Patient Safety, Communication about Error, Communication Openness, Reporting Patient Safety Events, Hospital Management Support for Patient Safety, and Handoffs and Information Exchange. Agreement items were rated from 1 (Strongly disagree) to 5 (Strongly agree), and frequency items were rated from 1 (Never) to 5 (Always). Responses of “Does not apply/Don’t know,” if selected, were treated as missing and excluded from the calculation of the corresponding dimension score. No such responses or other item-level missing values were identified in the analytic sample. Negatively worded items identified in the AHRQ scoring documentation were reverse scored so that higher values consistently represented more favorable patient safety culture. For individual-level analyses, each dimension score was calculated as the mean of its valid items and retained the original 1–5 metric. A dimension score was calculated only when a respondent had at least two valid items and had completed at least 50% of the items in that dimension. Consequently, both items were required for Reporting Patient Safety Events, at least two of three items were required for three-item dimensions, and at least two of four items were required for four-item dimensions. The 10-dimension scores were analyzed separately. An overall HSOPSC total score was not calculated because HSOPSC 2.0 is a multidimensional instrument and AHRQ provides scoring guidance for its separate composite dimensions rather than a global total. Internal consistency reliability was assessed separately for each HSOPSC dimension. In the present sample, Cronbach’s alpha was 0.664 for Teamwork, 0.689 for Staffing and Work Pace, 0.678 for Organizational Learning–Continuous Improvement, 0.744 for Response to Error, 0.716 for Supervisor/Manager/Clinical Leader Support for Patient Safety, 0.764 for Communication about Error, 0.763 for Communication Openness, 0.765 for Hospital Management Support for Patient Safety, and 0.746 for Handoffs and Information Exchange. For the two-item Reporting Patient Safety Events dimension, the Spearman–Brown coefficient was 0.907.

#### 2.3.3. Brief Resilience Scale (BRS)

Psychological resilience was measured using the Brief Resilience Scale (BRS) [36], a 6-item instrument rated on a 5-point Likert scale ranging from 1 (Strongly disagree) to 5 (Strongly agree). The BRS has been translated into Greek and psychometrically evaluated in Greek adults, showing adequate internal consistency and evidence of construct validity [37]. Higher scores indicate greater resilience. In the present study sample, the BRS demonstrated very good internal consistency reliability (Cronbach’s α = 0.880).

#### 2.3.4. Job Satisfaction Index (JSI)

Job satisfaction was assessed using the six-item Job Satisfaction Index developed by Schriesheim and Tsui [38] and subsequently reproduced in full by Tsui et al. [39]. It covers satisfaction with the nature of work, immediate supervisor, coworkers, pay, promotion opportunities, and the overall job situation. Responses were recorded on a five-point Likert scale ranging from 1 (strongly disagree) to 5 (strongly agree). Item scores were summed up to produce a total score ranging from 6 to 30, with higher scores indicating greater overall job satisfaction. All items were positively worded, and no reverse scoring was required. The JSI was translated and culturally adapted following guidelines for cross-cultural adaptation of self-reported measures [40]. Two independent bilingual translators, one nursing academic and one professional translator, produced forward translations, which were reconciled by the research team. A third bilingual translator, blinded to the original instrument, completed the backtranslation. An expert committee of two nursing academics reviewed the versions for semantic and conceptual equivalence. The preliminary Greek version was cognitively tested with 10 nurses who were not included in the main study sample; interviews lasted approximately 5–10 min and assessed clarity and comprehensibility. Minor wording modifications were made based on their feedback. No separate pilot study was undertaken. In the present study sample, the JSI demonstrated good internal consistency reliability (Cronbach’s α = 0.751).

### 2.4. Ethics Approval of Research

The study was approved by the Scientific Council of the University General Hospital of Alexandroupolis (protocol No. 7/16-06-2025). All procedures complied with the ethical principles of the Declaration of Helsinki.

### 2.5. Statistical Analysis

Statistical analysis was performed using IBM SPSS Statistics version 29 (IBM Corp., Armonk, NY, USA). Descriptive statistics were used to summarize participants’ demographic characteristics and study variables. Categorical variables are presented as frequencies and percentages, whereas continuous variables are presented as means and standard deviations (SD) or medians as appropriate. Before analysis, the dataset was examined for item-level and variable-level missing data. All 263 participants had complete data for the demographic variables and the HSOPSC, BRS, and JSI items used in the analyses. Therefore, no data imputation or complete-case deletion was required, and all analyses were based on the full analytic sample of 263 participants. As no missing data were identified, the extent and pattern of missingness did not differ between nurses working in ISO-certified and non-ISO-certified departments. The distributions of continuous demographic and study variables were assessed using the Kolmogorov–Smirnov test. A statistically significant result at *p* < 0.05 was considered evidence of departure from normality. Non-normally distributed demographic variables were summarized using medians and interquartile ranges and compared between groups using the Mann–Whitney U test. For the adjusted linear regression models, the distribution and behavior of the residuals were additionally evaluated using normal probability plots and residual-versus-fitted plots. Internal consistency reliability was assessed using Cronbach’s alpha for the BRS, the JSI, and each three- or four-item HSOPSC dimension. For the two-item Reporting Patient Safety Events dimension, reliability was estimated using the Spearman–Brown coefficient. Differences between nurses working in ISO-certified and non-ISO-certified departments were examined using independent-samples *t*-tests. When the assumption of homogeneity of variances was violated, Welch’s *t*-test was applied. Effect sizes were calculated using Hedges’ *g* and interpreted according to conventional criteria as small (0.20), medium (0.50), and large (0.80). Mean differences (MD), 95% confidence intervals (95% CI), test statistics, degrees of freedom (df), *p*-values, and effect sizes were reported for all comparisons. Spearman’s correlation analysis was conducted to ex-amine the relationships between patient safety culture, psychological resilience, and job satisfaction. Categorical variables were compared using chi-square tests, and non-normally distributed continuous demographic variables were compared using Mann–Whitney U tests. Adjusted linear regression models were performed as sensitivity analyses to examine the association between ISO certification and study outcomes after controlling for gender, age, educational level, department type, total professional experience, and years in the current department. Before interpreting the regression models, multicollinearity was assessed using tolerance and variance inflation factor statistics. Standardized residuals, leverage values, and Cook’s distances were examined to identify potentially influential observations. Heteroscedasticity-robust standard errors were used. Statistical significance was set at *p* < 0.05 (two-tailed) for all analyses. Because no formal a priori sample-size calculation was performed, an achieved-sample sensitivity analysis was conducted to provide a quantitative justification of the available sample rather than calculating retrospective observed power. With 165 nurses in ISO-certified departments and 98 nurses in non-ISO-certified departments, a two-sided α of 0.05, and 80% power, the achieved sample could detect a standardized between-group difference of approximately Cohen’s d = 0.36 (Hedges’ *g* ≈ 0.36) under the assumption of independent observations. This corresponds to a small-to-moderate standardized difference; effects smaller than this may not have been detected. Although nurses were recruited from 29 departments, department identifiers linking individual participants to their departments were not retained in the analytic dataset. Consequently, intraclass correlation coefficients, multilevel models, department-clustered standard errors, and a cluster-adjusted minimum detectable effect estimate could not be calculated. The reported regression coefficients therefore represent nurse-level mean differences associated with department certification status. Because within-department correlation could not be modeled, the conventional inferential estimates should be regarded as exploratory; heteroscedasticity robust standard errors address non-constant variance but do not correct dependence among nurses from the same department.

## 3. Results

A total of 263 nurses participated in the present study. The demographic characteristics of the participants are presented in Table 1. The majority of participants were female (85.9%) and had tertiary education (77.9%). Most participants worked in internal departments (65.4%). The median age of participants was 48 years, the median total professional experience was 16 years, and the median years of experience in the current department were 8 years. ISO-certified and non-ISO-certified departments did not differ significantly by gender, educational level, or age. However, the distribution of department type differed significantly between groups, with internal departments being more frequent among ISO-certified units and non-internal departments being more frequent among non-ISO-certified units. Nurses in ISO-certified departments also had significantly longer total professional experience and longer experience in their current department.

Reliability and descriptive statistics of study variables are presented in Table 2. Reliability coefficients for the HSOPSC dimensions ranged from 0.664 to 0.907. The BRS showed very good internal consistency (Cronbach’s α = 0.880), and the JSI showed acceptable internal consistency (Cronbach’s α = 0.751). Among the HSOPSC dimensions, the highest mean scores were observed for Supervisor/Manager/Clinical Leader Support for Patient Safety and Communication about Error, whereas the lowest mean scores were observed for Staffing and Work Pace and Organizational Learning–Continuous Improvement. The mean resilience score was 3.50 ± 0.74, while the mean job satisfaction score was 19.03 ± 4.01 (Table 2).

Comparisons between nurses working in ISO-certified and non-ISO-certified departments are presented in Table 3. Significant differences were observed in selected HSOPSC dimensions, although most dimensions did not differ significantly between the groups. Nurses working in ISO-certified departments reported significantly higher scores in Staffing and Work Pace compared with nurses working in non-ISO-certified departments. They also reported significantly higher scores in Reporting Patient Safety Events, suggesting more favorable perceptions of patient safety event reporting practices in ISO-certified departments. In contrast, nurses working in ISO-certified departments reported significantly lower scores in Handoffs and Information Exchange. Overall, the three statistically significant unadjusted differences were modest in standardized magnitude: Staffing and Work Pace (*g* = 0.40), Reporting Patient Safety Events (*g* = 0.37), and Handoffs and Information Exchange (*g* = −0.45); all remained statistically significant after Benjamini–Hochberg correction. The negative difference for Handoffs and Information Exchange indicates less favorable perceptions of information transfer and continuity of care among nurses working in ISO-certified departments. No statistically significant unadjusted differences were observed in psychological resilience or job satisfaction. These unadjusted findings were considered separately from the covariate-adjusted regression results reported below.

Most HSOPSC dimensions were positively correlated with both psychological resilience and job satisfaction. This pattern indicates that nurses reporting more favorable perceptions of specific patient-safety-culture domains also tended to report higher resilience and greater job satisfaction. The strongest dimension-level correlations with resilience were observed for Organizational Learning–Continuous Improvement (ρ = 0.420, *p* < 0.001), Teamwork (ρ = 0.412, *p* < 0.001), Hospital Management Support for Patient Safety (ρ = 0.402, *p* < 0.001), and Communication about Error (ρ = 0.399, *p* < 0.001). The strongest dimension-level correlations with job satisfaction were observed for Teamwork (ρ = 0.469, *p* < 0.001), Response to Error (ρ = 0.425, *p* < 0.001), Staffing and Work Pace (ρ = 0.418, *p* < 0.001), and Hospital Management Support for Patient Safety (ρ = 0.391, *p* < 0.001). Supervisor/Manager/Clinical Leader Support for Patient Safety was not significantly correlated with resilience (ρ = 0.043, *p* = 0.491), although it was positively correlated with job satisfaction (ρ = 0.377, *p* < 0.001). Psychological resilience was also positively correlated with job satisfaction (ρ = 0.280, *p* < 0.001) (Table 4).

No problematic multicollinearity was identified, with variance inflation factors ranging from 1.038 to 2.818 and tolerance values ranging from 0.355 to 0.963. Visual inspection of residual-versus-fitted and normal probability plots showed mild departures associated with the bounded outcome distributions but no severe pattern that precluded interpretation of the models. Heteroscedasticity-robust standard errors were therefore retained. The maximum Cook’s distance across all models was 0.077, indicating that no single observation exerted undue influence on the regression estimates.

Because the groups differed significantly in department type, total professional experience, and years in the current department, these characteristics were considered potential confounders and included in the adjusted regression models; gender, age, and educational level were also included. After adjustment, working in an ISO-certified department was associated with higher Staffing and Work Pace (B = 0.246, 95% CI [0.111, 0.381], *p* < 0.001), Reporting Patient Safety Events (B = 0.335, 95% CI [0.039, 0.632], *p* = 0.027), Supervisor/Manager/Clinical Leader Support (B = 0.221, 95% CI [0.062, 0.380], *p* = 0.006), and job satisfaction (B = 1.310, 95% CI [0.393, 2.227], *p* = 0.005), and with lower Handoffs and Information Exchange (B = −0.375, 95% CI [−0.542, −0.208], *p* < 0.001) and Hospital Management Support (B = −0.250, 95% CI [−0.425, −0.074], *p* = 0.005). No adjusted association with psychological resilience was observed (Table 5). After Benjamini–Hochberg correction across the 12 adjusted outcomes, the associations with Staffing and Work Pace, Handoffs and Information Exchange, Supervisor/Manager/Clinical Leader Support, Hospital Management Support, and job satisfaction remained statistically significant, whereas the association with Reporting Patient Safety Events did not. The unadjusted difference in job satisfaction was 0.612 points and was not statistically significant (*p* = 0.189), whereas the adjusted model estimated a difference of 1.310 points on the 6–30 JSI scale (95% CI [0.393, 2.227], *p* = 0.005, q = 0.014). This pattern indicates that the estimated association was sensitive to adjustment for measured participant and department characteristics.

## 4. Discussion

The present study identified a selective, modest, and mixed pattern of nurse-reported differences between ISO-certified and non-ISO-certified departments. In the unadjusted comparisons, nurses in ISO-certified departments reported more favorable Staffing and Work Pace and Reporting Patient Safety Events scores but less favorable Handoffs and Information Exchange scores, whereas psychological resilience and job satisfaction did not differ significantly. After adjustment, ISO-certified department status was positively associated with Staffing and Work Pace, local supervisory support, and job satisfaction and negatively associated with Handoffs and Information Exchange and hospital-level management support. The adjusted association with Reporting Patient Safety Events did not remain statistically significant after false-discovery-rate correction, and no association with psychological resilience was observed. Overall, these findings do not indicate a uniformly stronger patient safety culture or consistently more favorable nurse-related outcomes in ISO-certified departments.

Within the adapted Structure–Process–Outcome framework, ISO certification should be understood as a structural organizational characteristic rather than as an intervention that necessarily produces favorable outcomes [5]. Formal procedures, documented responsibilities, audits, corrective-action systems, and performance monitoring may contribute to clearer accountability, greater visibility of reporting processes, and more structured local supervisory practices. However, their effects depend on leadership commitment, staff engagement, organizational readiness, implementation maturity, and integration into everyday clinical work [8,9,10,11]. Certification may therefore strengthen selected organizational processes without resolving broader constraints related to workload, staffing, communication, coordination, or institutional support. This provides a plausible explanation for the selective rather than uniformly favorable pattern observed in the present study.

The contrasting associations with local supervisory support and hospital-level management support further suggest that quality-management arrangements may operate differently across organizational levels. More favorable perceptions of Supervisor/Manager/Clinical Leader Support may reflect clearer departmental responsibilities, more visible monitoring, or more structured day-to-day supervisory practices. In contrast, Hospital Management Support may reflect broader experiences of institutional communication, resource allocation, psychological safety, and organizational responsiveness [11,34]. Local supervisory support and hospital-level management support should therefore not be considered interchangeable. However, leadership practices, communication between organizational levels, and nurses’ direct experiences of certification procedures were not measured, and this interpretation remains provisional.

The less favorable Handoffs and Information Exchange scores also indicate that standardization within departments may not ensure effective coordination across professional, departmental, and technological boundaries. Handoffs and transitions are recurrently vulnerable aspects of care, particularly when staffing pressures, interruptions, information overload, insufficient training, and difficulties distinguishing essential from nonessential information are present [41,42,43,44]. Structured approaches such as I-PASS and SBAR may improve information transfer, but their effectiveness depends on implementation quality, staff preparation, protected time, compatible information systems, and adaptation to local workflows [45,46,47]. It is also possible that nurses in certified departments had greater exposure to quality standards, audits, and formal expectations and consequently evaluated handoff practices more critically. Because awareness of standards and objective handoff performance were not measured, lower scores should not be interpreted as evidence that certification caused poorer handoffs.

The differences in department type, total professional experience, and years of experience in the current department should also be considered. Adjustment for these characteristics altered some estimates, particularly the association with job satisfaction, suggesting that group composition may have masked or modified the crude associations. Although the adjusted job-satisfaction difference was statistically significant, its practical importance remains uncertain, and most of the variation in job satisfaction remained unexplained. Residual confounding by leadership style, staffing, workload, patient case mix, team functioning, organizational climate, and certification maturity cannot be excluded. The findings should therefore be interpreted as adjusted nurse-level associations rather than as evidence of an independent certification effect.

The absence of an association with resilience and the sensitivity of the job-satisfaction results in adjustment supporting a distinction between specific organizational processes and broader nurse-related outcomes. Resilience and job satisfaction are shaped by multiple individual, interpersonal, professional, and organizational factors, including coping resources, social support, autonomy, recognition, leadership, staffing, workload, scheduling, team relationships, and work–life balance [20,22,23,24,25,26,27,28,29,30,31,32,33]. Certification status alone may therefore be too distal and nonspecific to explain substantial variation in these outcomes. Nevertheless, most patient-safety-culture dimensions were positively correlated with resilience and job satisfaction. This suggests that nurses’ immediate experiences of teamwork, organizational learning, communication, responses to error, staffing, and management support may be more closely related to their well-being than formal certification status itself [28,31,32,33,34,48]. The cross-sectional design, however, did not permit examination of causal or mediating pathways.

The national and regional context may also influence these relationships. Southern European healthcare systems are heterogeneous and should not be treated as a single organizational model. Nevertheless, public-sector administration in the region has historically been characterized by relatively formalized rules, hierarchical decision-making, and challenges in translating centrally introduced reforms into consistent organizational practice [49]. In Greece, resource and workforce constraints may limit the capacity of hospital departments to integrate quality-management requirements into everyday care, even when formal certification structures are present [50]. Evidence from Greek public hospitals also indicates that organizational culture and differing administrative logics across hierarchical levels may influence the implementation of standardized reforms [51]. These characteristics may help explain why certification was associated with selected departmental processes but not with uniformly favorable outcomes. The findings may therefore be most transferable to public hospitals operating under comparable administrative, staffing, and resource conditions and should not be generalized automatically to countries with different governance structures, workforce capacity, professional autonomy, or quality-management traditions.

Effect magnitude and measurement level should also guide interpretation. Most unadjusted differences were trivial or small, while only Staffing and Work Pace, Reporting Patient Safety Events, and Handoffs and Information Exchange reached small-to-moderate magnitudes. Modest differences may still identify processes requiring organizational attention when they concern recurrent activities such as workload organization, incident reporting, and transfer of clinical information. However, the study assessed nurses’ perceptions rather than objective reporting rates, handoff failures, adverse events, or patient outcomes. It therefore cannot be determined whether the observed differences corresponded to measurable differences in safety performance.

Overall, ISO-certified department status appears to represent one element of a broader organizational context rather than a standalone determinant of patient safety culture, psychological resilience, or job satisfaction. Formal certification should be complemented by initiatives addressing communication across organizational boundaries, consistency between departmental and hospital-level leadership, teamwork, staffing and workload, handoff systems, professional development, and staff well-being. Future multicenter and longitudinal research should assess certification scope, duration, and implementation maturity; retain department-level identifiers; apply multilevel analyses; and incorporate objective patient safety indicators.

### Limitations

Several limitations should be considered. First, the cross-sectional design does not allow for causal conclusions regarding the relationships among ISO certification, patient safety culture, psychological resilience, and job satisfaction. Second, data were collected using self-report questionnaires; therefore, social-desirability and recall biases may have influenced participants’ responses. Furthermore, objective patient-safety indicators, such as incident-reporting rates, handoff failures, adverse events, and patient outcomes, were not assessed. Consequently, it cannot be determined whether the observed differences in nurses’ perceptions corresponded to measurable differences in patient-safety performance. In addition, three HSOPSC dimensions—Teamwork, Staffing and Work Pace, and Organizational Learning—Continuous Improvement showed only moderate internal consistency in the present sample. This may have reduced measurement precision and attenuated the observed associations or between-group differences involving these dimensions. Findings related to these subscales should therefore be interpreted with caution. Third, the study was conducted in a single tertiary hospital in Northern Greece, which may limit the generalizability of the findings to other healthcare settings, regions, or healthcare systems. Although Southern European healthcare systems are heterogeneous, the findings may be more transferable to public hospitals operating under broadly comparable administrative, staffing, and resource conditions; their applicability to systems with different governance structures, workforce capacity, professional autonomy, or quality-management traditions should be considered cautiously. In addition, departments were purposively rather than randomly selected, and nurses within those departments were recruited using convenience sampling. This non-probability sampling strategy may have introduced selection bias because the included departments and participating nurses may differ systematically from departments and nurses who were not represented. The selected departments may therefore not be representative of other departments within the same hospital or of departments in other healthcare institutions, further limiting the external validity of the findings. The significant difference in department type between the ISO-certified and non-ISO-certified groups further indicates that the groups were not fully comparable at baseline. Although department type and other measured characteristics were included in the adjusted analyses, residual selection bias and confounding by unmeasured organizational characteristics cannot be excluded. Fourth, no formal a priori sample-size calculation was performed, and the total number of eligible or invited nurses was not recorded; therefore, a response rate could not be calculated, and the possibility of nonresponse bias cannot be excluded. Although the achieved sample was sufficient to detect a standardized between-group difference of approximately d = 0.36 under an independent-observations assumption, smaller differences may not have been detected. In addition, ISO-certification status was a department-level exposure, whereas the outcomes and analyses were at the individual nurse level. Nurses working within the same department may share leadership, staffing, workflow, communication systems, and other organizational conditions, creating within-department correlations. Participant-level department identifiers were not retained in the analytic dataset; consequently, intraclass correlations, multilevel models, and department-clustered standard errors could not be calculated. The analyses therefore treated 263 nurses as independent observations even though the effective number of independent organizational exposure units was the number of participating departments. This may have produced standard errors that were too small and confidence intervals that were too narrow, increasing the possibility of false-positive findings. The adjusted associations should therefore be considered exploratory and require confirmation in studies designed and analyzed at multiple organizational levels. Fifth, detailed information regarding the specific ISO standard and version, certification scope, duration, recertification history, compliance, and maturity of implementation was not available for analysis. ISO certification was therefore treated as a binary department-level characteristic, although certified departments may have differed substantially in the duration, intensity, and quality of implementation. This limits comparisons with other certified settings and prevents determination of whether the observed associations varied according to quality-management-system maturity. Documentation burden, procedural complexity, compliance-related workload, and the quality of handoff practices were also not measured. Consequently, the proposed explanation for the less favorable Handoffs and Information Exchange scores in ISO-certified departments remains an untested hypothesis. Finally, potentially relevant organizational and individual factors, such as leadership style, staffing ratios, workload, burnout, organizational support, and workplace violence, were not examined. These limitations require cautious interpretation of the reported exploratory nurse-level associations.

## 5. Conclusions

In this exploratory nurse-level analysis, working in ISO-certified departments was associated with a selective and mixed pattern of reported outcomes, including more favorable perceptions of Staffing and Work Pace and Reporting Patient Safety Events, less favorable perceptions of Handoffs and Information Exchange, and contrasting adjusted associations with local supervisory and hospital-level management support. The unadjusted Reporting Patient Safety Events difference remained significant after correction, whereas the corresponding adjusted association did not. The observed differences were modest and cannot be attributed specifically to ISO certification because within-department clustering and potentially important organizational confounders were not addressed. Objective patient-safety outcomes were also not measured. Future studies should recruit sufficient numbers of comparable certified and non-certified departments and use multi-level analyses incorporating certification maturity, workflow characteristics, and objective safety indicators.

## Figures and Tables

**Table 1:** Demographic characteristics of the participants.

Characteristic	Total *N* = 263	ISO-Certified *n* = 165	Non-ISO-Certified *n* = 98	*p*-Value
Gender, *n* (%)				0.656
Male	37 (14.1)	22 (13.3)	15 (15.3)	
Female	226 (85.9)	143 (86.7)	83 (84.7)	
Educational level, *n* (%)				0.177
Secondary education	58 (22.1)	32 (19.4)	26 (26.5)	
Tertiary education	205 (77.9)	133 (80.6)	72 (73.5)	
Department type, *n* (%)				<0.001
Internal departments	172 (65.4)	126 (76.4)	46 (46.9)	
Non-internal departments	91 (34.6)	39 (23.6)	52 (53.1)	
Age, median [IQR]	48.0 [40.0, 55.0]	48.0 [42.0, 55.0]	48.5 [39.0, 52.0]	0.146
Total professional experience, median [IQR]	16.0 [5.0, 22.0]	18.0 [5.0, 22.0]	10.0 [5.0, 20.0]	0.004
Years in current department, median [IQR]	8.0 [3.0, 17.0]	10.0 [4.0, 18.0]	4.0 [2.0, 10.0]	<0.001

Note: Categorical variables were compared using chi-square tests. Continuous variables were compared using Mann–Whitney U tests because age and experience variables were non-normally distributed. Surgical and laboratory departments were merged into the non-internal department category.

**Table 2:** Reliability and descriptive statistics of study variables.

Variable	Reliability Coefficient	Mean ± SD	Median [IQR]
Teamwork	0.664	3.02 ± 0.72	3.0 [2.7, 3.7]
Staffing and Work Pace	0.689	2.44 ± 0.56	2.2 [2.0, 2.8]
Organizational Learning–Continuous Improvement	0.678	2.74 ± 0.75	2.7 [2.0, 3.3]
Response to Error	0.744	3.59 ± 0.68	3.5 [3.2, 4.0]
Supervisor/Manager/Clinical Leader Support	0.716	3.98 ± 0.67	4.0 [3.7, 4.3]
Communication about Error	0.764	3.95 ± 0.77	4.0 [3.7, 4.5]
Communication Openness	0.763	3.60 ± 0.72	3.7 [3.3, 4.0]
Reporting Patient Safety Events	0.907	3.81 ± 1.18	4.0 [3.0, 5.0]
Hospital Management Support	0.765	3.11 ± 0.74	3.0 [2.7, 3.7]
Handoffs and Information Exchange	0.746	3.71 ± 0.67	3.7 [3.3, 4.0]
Brief Resilience Scale	0.880	3.50 ± 0.74	3.7 [3.0, 4.0]
Job Satisfaction Index total	0.751	19.03 ± 4.01	19.0 [16.0, 22.0]

Note: Cronbach’s alpha coefficients are reported for the three- and four-item HSOPSC dimensions, the BRS, and the JSI. The Spearman–Brown coefficient is reported for the two-item Reporting Patient Safety Events dimension.

**Table 3:** Comparison of study variables between ISO-certified and non-ISO-certified departments.

Variable	ISO-Certified Mean (SD)	Non-ISO-Certified Mean (SD)	MD	95% CI	t (df)	*p*-Value	Hedges’ *g*
Teamwork	2.984 (0.788)	3.075 (0.595)	−0.091	[−0.260, 0.078]	−1.059 (246.354)	0.291	−0.126
Staffing and Work Pace	2.518 (0.597)	2.296 (0.453)	0.222	[0.094, 0.351]	3.407 (245.656)	0.001	0.404
Organizational Learning–Continuous Improvement	2.792 (0.803)	2.639 (0.652)	0.152	[−0.026, 0.331]	1.678 (236.846)	0.095	0.202
Response to Error	3.609 (0.722)	3.556 (0.597)	0.053	[−0.109, 0.215]	0.643 (234.139)	0.521	0.078
Supervisor/Manager/Clinical Leader Support	4.008 (0.706)	3.925 (0.603)	0.083	[−0.079, 0.245]	1.011 (229.445)	0.313	0.123
Communication about Error	3.947 (0.795)	3.952 (0.721)	−0.005	[−0.193, 0.184]	−0.051 (219.919)	0.959	−0.006
Communication Openness	3.661 (0.697)	3.510 (0.746)	0.150	[−0.033, 0.334]	1.619 (192.873)	0.107	0.210
Reporting Patient Safety Events	3.973 (1.168)	3.546 (1.160)	0.427	[0.134, 0.719]	2.878 (204.970)	0.004	0.365
Hospital Management Support	3.055 (0.734)	3.207 (0.739)	−0.153	[−0.338, 0.032]	−1.627 (202.684)	0.105	−0.207
Handoffs and Information Exchange	3.600 (0.669)	3.895 (0.638)	−0.295	[−0.458, −0.131]	−3.556 (211.680)	<0.001	−0.447
Brief Resilience Scale	3.501 (0.782)	3.486 (0.681)	0.015	[−0.166, 0.196]	0.159 (226.160)	0.874	0.020
Job Satisfaction Index total	19.255 (4.491)	18.643 (3.027)	0.612	[−0.303, 1.526]	1.317 (256.814)	0.189	0.152

Note: Welch’s *t*-test was used for group comparisons. MD = mean difference calculated as ISO-certified minus non-ISO-certified departments; CI = confidence interval.

**Table 4:** Associations between patient safety culture, resilience, and job satisfaction.

Patient Safety Culture Dimension	BRS, ρ	*p*-Value	JSI, ρ	*p*-Value
Teamwork	0.412	<0.001	0.469	<0.001
Staffing and Work Pace	0.228	<0.001	0.418	<0.001
Organizational Learning–Continuous Improvement	0.420	<0.001	0.334	<0.001
Response to Error	0.252	<0.001	0.425	<0.001
Supervisor/Manager/Clinical Leader Support for Patient Safety	0.043	0.491	0.377	<0.001
Communication about Error	0.399	<0.001	0.360	<0.001
Communication Openness	0.296	<0.001	0.358	<0.001
Reporting Patient Safety Events	0.288	<0.001	0.158	0.010
Hospital Management Support for Patient Safety	0.402	<0.001	0.391	<0.001
Handoffs and Information Exchange	0.259	<0.001	0.328	<0.001

Note: BRS = Brief Resilience Scale; JSI = Job Satisfaction Index; ρ = Spearman’s correlation coefficient. The correlation between BRS and JSI was ρ = 0.280, *p* < 0.001.

**Table 5:** Adjusted linear regression models examining associations between ISO-certified department status and study outcomes.

**Panel A. ISO-Certification Status and Categorical Covariates**					
**Outcome**	**ISO-Certified Department, B (*p*)**	**BH-Adjusted q-Value**	**Female Gender, B (*p*)**	**Tertiary Education, B (*p*)**	**Non-Internal Department, B (*p*)**
Teamwork	−0.075 (0.430)	0.516	−0.153 (0.130)	0.359 (0.001)	0.193 (0.047)
Staffing and Work Pace	0.246 (<0.001)	<0.006	0.024 (0.758)	0.190 (0.019)	0.168 (0.030)
Organizational Learning–Continuous Improvement	0.186 (0.061)	0.105	−0.239 (0.099)	−0.121 (0.288)	0.125 (0.253)
Response to Error	−0.016 (0.859)	0.859	0.155 (0.175)	0.004 (0.970)	0.006 (0.948)
Supervisor/Manager/Clinical Leader Support for Patient Safety	0.221 (0.006)	0.014	0.200 (0.092)	−0.269 (0.006)	0.289 (<0.001)
Communication about Error	−0.074 (0.403)	0.516	−0.255 (0.032)	−0.165 (0.063)	−0.057 (0.530)
Communication Openness	0.170 (0.072)	0.108	−0.249 (0.047)	−0.071 (0.490)	0.069 (0.484)
Reporting Patient Safety Events	0.335 (0.027)	0.054	−0.480 (0.018)	−0.421 (0.006)	−0.408 (0.012)
Hospital Management Support for Patient Safety	−0.250 (0.005)	0.014	−0.073 (0.539)	0.062 (0.637)	−0.214 (0.021)
Handoffs and Information Exchange	−0.375 (<0.001)	<0.006	0.384 (0.006)	−0.282 (0.005)	−0.379 (<0.001)
Brief Resilience Scale	0.047 (0.618)	0.674	−0.403 (<0.001)	−0.056 (0.644)	0.187 (0.056)
Job Satisfaction Index total	1.310 (0.005)	0.014	0.097 (0.913)	−0.098 (0.886)	2.551 (<0.001)
**Panel B. Continuous Covariates and Model Fit**					
**Outcome**	**Age, B (*p*)**	**Total Professional Experience, B (*p*)**	**Years in Current Department, B (*p*)**	**R^2^**	**Adjusted R^2^**
Teamwork	0.011 (0.070)	0.004 (0.554)	−0.006 (0.429)	0.090	0.065
Staffing and Work Pace	−0.005 (0.319)	0.007 (0.315)	−0.001 (0.900)	0.094	0.069
Organizational Learning–Continuous Improvement	−0.000 (0.993)	0.014 (0.133)	−0.009 (0.293)	0.048	0.021
Response to Error	0.002 (0.785)	0.016 (0.060)	0.001 (0.893)	0.074	0.048
Supervisor/Manager/Clinical Leader Support for Patient Safety	0.019 (<0.001)	−0.012 (0.054)	−0.008 (0.174)	0.149	0.126
Communication about Error	0.027 (<0.001)	0.019 (0.015)	−0.015 (0.027)	0.217	0.196
Communication Openness	0.013 (0.043)	0.013 (0.098)	−0.017 (0.028)	0.088	0.063
Reporting Patient Safety Events	0.019 (0.054)	0.012 (0.259)	−0.020 (0.103)	0.139	0.116
Hospital Management Support for Patient Safety	0.009 (0.224)	0.015 (0.082)	−0.012 (0.101)	0.073	0.047
Handoffs and Information Exchange	−0.007 (0.292)	0.012 (0.060)	−0.013 (0.011)	0.209	0.187
Brief Resilience Scale	0.010 (0.187)	0.000 (0.975)	0.004 (0.596)	0.063	0.037
Job Satisfaction Index total	0.098 (0.030)	−0.091 (0.055)	0.052 (0.145)	0.120	0.096

Note. Values are unstandardized regression coefficients, B, with *p*-values in parentheses. Reference categories were non-ISO-certified department, male gender, secondary education, and internal department. Models were adjusted for gender, age, educational level, department type, total professional experience, and years in current department. Heteroscedasticity-robust standard errors were used. Benjamini–Hochberg-adjusted q-values were calculated for the ISO-certification coefficients across the 12 prespecified outcomes comprising the 10 HSOPSC dimensions, the Brief Resilience Scale, and the Job Satisfaction Index. BH = Benjamini–Hochberg.

## Data Availability

The data presented in this study is available on request from the corresponding author. The data is not publicly available due to privacy and ethical restrictions.

## Abbreviations

The following abbreviations are used in this manuscript:

HSOPSC	Hospital Survey on Patient Safety Culture
BRS	Brief Resilience Scale
JSI	Job Satisfaction Index
ISO	International Organization for Standardization
SD	Standard deviation
IQR	Interquartile range
CI	Confidence interval
MD	Mean difference
SPSS	Statistical Package for Social Sciences
